# Dynamism in paintings and photographs: how the power of diagonals in art overcomes the oblique effect in visual perception

**DOI:** 10.64898/2026.07.22.739875

**Published:** 2026-08-06

**Authors:** Serena Castellotti, Elvio Blini, Maria Del Viva, Qasim Zaidi

## Abstract

Artists and photographers use diagonals to convey dynamism, tension and instability in naturalistic and abstract images. However, the oblique effect in visual perception refers to a reduced sensitivity to oblique compared to cardinal (vertical/horizontal) orientations, linked to anisotropy in the distribution of preferred orientations of primary visual cortex neurons, which reflect the statistical distribution of edge orientations in natural images. Distal diagonals generically project onto oblique orientations on the retina, creating a conundrum between their perceptual salience in art and their reduced sensitivity in vision.

We conjectured that global spatial configurations could shape perceived dynamism overriding local orientations. Inspired by van Doesburg’s 1929 Arithmetical Composition, we created 60 distinct linear configurations of four identically-shaped rhombi each, in which global and local-edge orientations were independently manipulated to be cardinal or oblique, and measured their perceived dynamism with behavioral, psychophysical, and psychophysiological methods.

First, 250 observers rated oblique configurations as more dynamic, tridimensional, and unstable than cardinal configurations, with wedge shapes and element spacing enhancing dynamism. Second, pairwise comparisons (50 observers) yielded a perceptual dynamism scale that confirmed the dominant role of global orientations. Third, pupillometry (35 observers) showed that pupil dilation correlates systematically with subjective dynamism ratings.

Perceived dynamism in static images is thus driven primarily by the orientation and shape of spatial configurations, indicating the importance of estimating orientations and shapes of multi-element objects beyond decoding local orientations. By linking visual structure to emotional perceptual experience, this investigation provides empirically grounded principles for creating dynamic percepts in visual art.

**SIGNIFICANCE STATEMENT:**

Diagonals have been used since Michelangelo to create dynamic compositions in representative and abstract art and photography, but paradoxically humans have been shown to be less sensitive to oblique orientations like those projected on the retina by diagonal edges.

We resolve this conflict by distinguishing between global configurations versus local edges and showing through behavioral, psychophysical, and psychophysiological experiments that the global orientation and shape of a multi-element configuration has a much larger effect on perceived dynamism than do the edge orientations of its elements.

Neural estimation of orientations and shapes of multi-element configurations is thus essential for image understanding. Our results provide perceptually grounded bases for using diagonal configurations with wedge shapes to create dynamic percepts in visual art.

## Full Text Availability

The license terms selected by the author(s) for this preprint version do not permit archiving in PMC. The full text is available from the preprint server.

